# Improving Bitumen Properties with Chitosan: A Sustainable Approach to Road Construction

**DOI:** 10.3390/molecules30051170

**Published:** 2025-03-05

**Authors:** Paolino Caputo, Cesare Oliviero Rossi, Pietro Calandra, Debora Policastro, Eugenia Giorno, Nicolas Godbert, Iolinda Aiello

**Affiliations:** 1 Department of Chemistry and Chemical Technologies, Università della Calabria, Via P. Bucci, Cubo 14/D, 87036 Rende, CS, Italy; paolino.caputo@unical.it; 2 CNR-ISMN, National Research Council, Institute for the Study of Nanostructured Materials, Strada Provinciale 35 D n.9, 00010 Montelibretti, RM, Italy; 3 MAT-InLAB, LASCAMM CR-INSTM, Unità INSTM della Calabria, Dipartimento di Chimica e Tecnologie Chimiche, Università della Calabria, 87036 Arcavacata di Rende, CS, Italy; debora.policastro@unical.it (D.P.); eugenia.giorno@unical.it (E.G.); nicolas.godbert@unical.it (N.G.); iolinda.aiello@unical.it (I.A.); 4 LPM-Laboratorio Preparazione Materiali, Star-Lab, Università della Calabria, 87036 Arcavacata di Rende, CS, Italy; 5 CNR NANOTEC, UOS Rende c/o Dipartimento di Fisica, Università della Calabria, Via P. Bucci 33C, 87036 Arcavacata di Rende, CS, Italy

**Keywords:** bitumen, chitosan, thermal stability, boiling test

## Abstract

This research explores the utilization of chitosan, a naturally derived biopolymer, as an innovative additive in bitumen for road construction. The experimental procedure for incorporating chitosan into bitumen, in agreement with its thermal stability, is described. Four different types of chitosan (two different degrees of deacetylation: >75 and >90% in free amine groups; molecular weight ranging from 100 to 800 kD) have been considered. Each chitosan was added to a bitumen at 1, 3, 6 wt%, and the mechanical characteristics were tested by dynamic shear rheology with the aim of testing the thermal stability of modified bitumen. An increase in the gel-to-sol temperature transition was generally found in the presence of chitosan, suggesting enhanced resistance to deformation under traffic loads. The most marked effect was obtained for chitosan with a molecular weight of 310,000–375,000 kD and with a deacetylation degree ≥75% (free amine groups). In addition, it was found that chitosan can slow down the oxidative aging of bitumen, especially when chitosan with high molecular weight (600,000–800,000 kD) and with a deacetylation degree >90% (free amine groups) was used. This further finding suggests that chitosan can potentially extend the final road pavement life.

## 1. Introduction

Bitumen is a black, semi-solid or liquid, viscous material that is produced in distillation of crude oil in the refining process of petroleum. Bitumen has seen extensive use for decades now in roofing, pavement engineering, and numerous other industrial applications. Viscoelasticity and mechanical properties of bitumen are key parameters in influencing its behavior across the various applications [1,2,3]. 

However, unmodified bitumen often fails to meet the required engineering standards, making modifications essential and necessary to enhance its mechanical strength and adhesive properties. Furthermore, with the significant rise in traffic speed and load, the durability of asphalt pavements is strongly affected, leading to a reduction in their lifespan. There are many efforts from researchers to investigate additives to compensate or improve the quality of the bitumen. The additives are used in bitumen and asphalt mixtures technologies to improve their properties: modifiers [4], fillers [5], rejuvenators [6], emulsifiers/surfactants [7] are just examples of widely increasing use of additives.

In this ambit, chitosan (CS) is a natural polymer found in the exoskeletons of crustaceans like shrimp and crabs that can be easily extracted from the waste shells of crustaceans derived from seafood processing industries [8,9]. It is biodegradable and biocompatible. It has also antimicrobial properties, thus being valuable and versatile material across a wide range of industries and applications [10,11].

In the field of bitumen technology, CS has been little investigated, even if it has been already shown that it can modify the properties of bitumen and improve its resistance to water-induced degradation [12].

**Figure 1:**
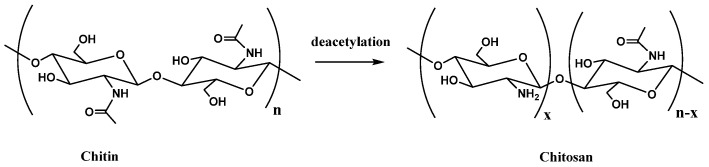
Chitin and chitosan molecular structures. Note that the **x** value in chitosan represents the deacetylation grade of chitosan.

From the chemical point of view, CS is a partially or fully deacetylated form of chitin consisting of β-(1→4) 2-acetamido-2-deoxy-D-glucose units (see Figure 1 for molecular structures of chitin and chitosan) where primary amine group is responsible for its unique properties [13]. For this reason, the deacetylation degree is another important characteristic that has to be taken into account. Being an amphiphilic polyelectrolyte, it can combine both electrosteric and viscosifying stabilization mechanisms [14], thus stabilizing oil and water emulsion systems [14,15]. For this reason, CS has been first investigated as a good emulsifier for bitumen emulsions [16].

Special treatments and/or interactions with other molecules can give synergic effects in bitumen: as an example, the graphene oxide–glutaraldehyde cross-linked CS composite has been used in combination with a styrene–butadiene–styrene modified asphalt (SBSMA) binder to improve the viscosity, elasticity, thermal stability and rutting resistance performance of the modified asphalt [17,18]. More recently, the crosslinking of CS with epichlorohydrin has shown that the cross-linked CS enhances asphaltene separation, reduces viscosity and improves cohesion energy and performances with limestone and granite [12]. Apart from these studies, to the best of our knowledge, no deeper investigation has been performed onto the modification of the rheological and antioxidant properties of bitumen in the presence of CS.

Despite these positive findings, few studies have been reported on the modification of bitumen with CS. Even though a few studies have been carried out on its application as an emulsifier or a component in composite blends, the effect of CS on the rheological and antioxidant properties of bitumen is still unclear. To the best of our knowledge, there is no comprehensive investigation on how CS influences the viscoelasticity of bitumen, antioxidative aging resistance, and long-term performance in different conditions. Furthermore, the nature of these changes must be understood better. Closure of such gaps is important in terms of being able to utilize the full potential of CS in bitumen applications. The principal objective of the present work is therefore to explore the employment of CS as a rheological modifier and antioxidant in bitumen formulations.

## 2. Results

### 2.1. Thermal Stabilities of the Four CS Polymers

**Figure 2:**
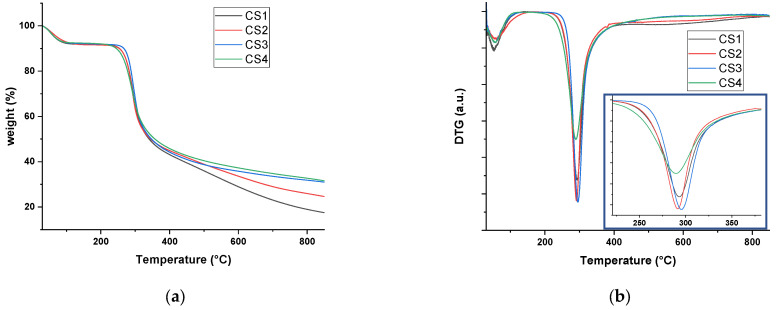
(**a**) TGA thermograms of the CS polymers (**CS1–4**); (**b**) relative DTG curves; in inset, detail of the DTG curves at ca. 290 °C.

Since the blending of CS with bitumen takes place at around 150 °C (see Section 3.2 of the experimental section) the thermal stability of all the four investigated CS polymers was tested by thermogravimetric analysis (TGA). The thermograms are reported in Figure 2a together with the derivative curves (DTG) reported in Figure 2b.

All polymers are characterized by a first initial weight loss that is owed to their intrinsic dehydration, i.e., the loss of water molecules loosely bonded to the polymer chains. The second and more intense loss is characterized by a maximum rate situated at ca. 290 °C, as clearly observed on the DTG curves (Figure 2b) by a marked minimum. This degradation step is relative to the deamination, deacetylation and depolymerization of CS [19] and is, indeed, accompanied by the release of H_2_O, NH_3_, CO, CO_2_ and CH_3_COOH [20]. Finally, a last thermal event registered as a slow and continuous rate at temperatures over 400 °C corresponds to the residual decomposition reactions, such as dehydrogenation with production of CH_4_ [20]. Within the series **CS1** to **CS4**, only slight differences are encountered on the temperature of the second thermal decomposition event; for **CS1**, the maximum rate temperature is recorded at 293 °C, while it is 291 °C for **CS2**, 296 °C for **CS3** and 289 °C for **CS4** (see the inset of Figure 2b). The main difference between the thermograms is instead recorded during the third and last thermal event, with a more pronounced decrease in weight loss being recorded, as expected, for the two **CS1** and **CS2** polymers characterized by a lower deacetylation grade (over 75%) with respect to **CS3** and **CS4**, which are characterized by a deacetylation grade over 90%. Accordingly, to these thermograms, it can be concluded that, during mixing at the temperature of 150 °C with bitumen, no degradation of the CS polymers can be expected.

**Figure 3:**
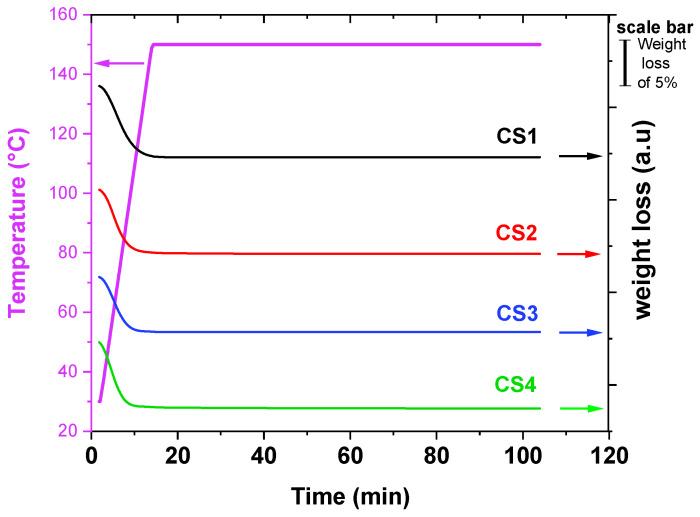
Recorded isotherms at 150 °C for all CS polymers (**CS1–4**). Note that arrows are indicating the y-axis relative to the corresponding plot.

In any case, to ascertain the CS integrity over time at high temperatures, the thermal stability of the employed CS polymers was also probed as a function of time at the fixed temperature of 150 °C by recording the weight at isotherm conditions. The results are reported in Figure 3.

All isotherms curves (Figure 3) display a quick weight loss during the heating step (first 10 min) due to the water release during heating. Temperature was then maintained at 150 °C for further 95 min, and no other process was detected for the duration of the monitoring. As a result of this TGA investigation, it can be concluded that all the CS polymers considered for the present studies can safely be blended with bitumen at 150 °C with no degradation.

### 2.2. Rheological Modifying Effect

**Figure 4:**
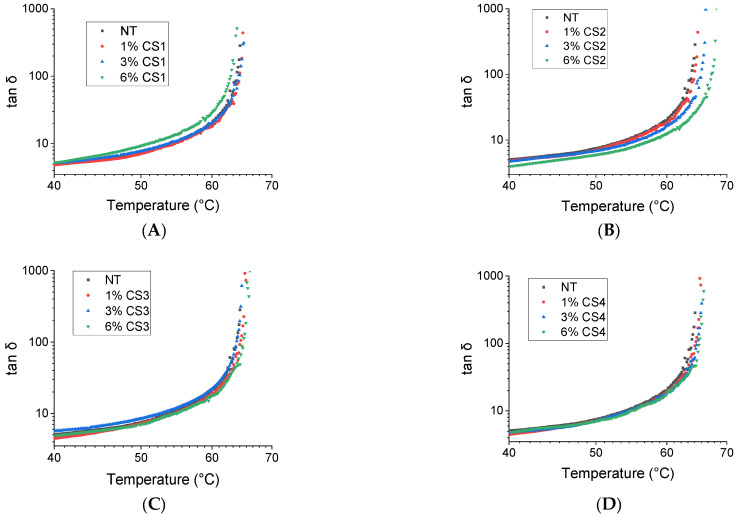
Tan δ as a function of temperature at various CS content: panel (**A**) (**CS1**, molecular weight 50,000–190,000 kD, with a deacetylation degree ≥75%, free amine groups); panel (**B**) (**CS2**, molecular weight 310,000–375,000 kD with a deacetylation degree ≥75%, free amine groups); panel (**C**) (**CS3**, molecular weight 100,000–300,000 kD with a deacetylation degree >90% free amine groups); panel (**D**) (**CS4**, molecular weight 600,000–800,000 kD with a deacetylation degree >90%, free amine groups).

Before showing the results of the dynamic rheological analysis, it must first be clarified that a good bitumen modifier improves the physico-chemical property of the bitumen, increasing the transition temperature of the binder [21]. If this is achieved, the road pavement will show an improvement in the performance at high temperatures. The results of the dynamic rheological analysis, carried out on paving bitumen before and after addition of all **CS1–4** at 1, 3 and 6 wt%, are shown in Figure 4.

In time-cure tests, the tangent delta (tan δ = G″/G′), defined as the ratio of the loss modulus (G″) to the storage modulus (G′), is measured while applying a temperature ramp at a constant heating rate of 1 °C/min and a fixed frequency of 1 Hz. In these assessments, the real part of the complex modulus G′ represents the in-phase component, reflecting the reversible elastic energy, while the imaginary part G″ is corresponding to the out-of-phase component, indicating the irreversible dissipation of mechanical energy. [22]. As the temperature rises, the samples gradually soften, leading to a continuous decrease in G′. When the molecular relaxation rate becomes sufficiently high, allowing the material to respond to mechanical deformation, bitumen can be therefore considered as almost a true Newtonian fluid. Consequently, G′ approaches zero, causing tan δ to diverge. This results in a true gel-to-sol transition, with the corresponding temperature (T*) serving as an indicator of the sample’s thermal stability. An effective rheological modifier therefore enhances this transition temperature. The value of T* is reported in Table 1 for the various samples (see second column). It can be seen that the samples modified with **CS1–4** have similar rheological profiles, showing all an increase in transition temperature due to the presence of CS.

The sample **CS2** gives more marked changes in T*, as can be seen from Table 1 and even by a visual inspection of Figure 3. The effect can be evidenced even at a CS content of 3 wt% and obviously increases progressively with the CS content, reaching the highest value of the whole set of samples at 6 wt%. Evidently **CS2** possesses the optimized combination of an appropriate molecular weight and deacetylation degree for modifying the bitumen at high temperatures. Specifically, some justification can be hypothesized by considering the peculiar molecular composition of the bitumen, which includes a wide variety of apolar molecules (making part of the so-called maltene fraction), amphiphilic species (basically included in the fraction of resins) and the polar ones (i.e., asphaltenes), the latter being characterized by the presence of polar functional groups and heteroatoms. The CS molecules, whose molecular structure is recalled in Figure 1, possess (apart from hydroxyls and ethers) secondary acetamide and primary ammine groups, whose relative abundance is dictated by the deacetylation grade of the specific CS. The fact that **CS2** can enhance T* more effectively than the other CSs (**CS1**, **CS3** and **CS4**) could be due to the fact that CS interacts efficiently with the polar species of the bitumen via its secondary amide group. It must be noted, in fact, that a CS with a lower deacetylation degree possesses more C=O groups which are able to interact with the polar groups of the bitumen and even to establish relatively strong H-bonds with them. In addition, the amide part can behave as a multi-dentate (even chelating) interacting agent. In addition, the high molecular weight of **CS2** ultimately facilitates optimal interactions between bitumen and CS, enhancing the solidity and stability of the bitumen–CS complexes. As a result, the overall supramolecular structure grip can better sustain the increase in temperature.

### 2.3. Antioxidant Effect

An agent is referred to as antioxidant if it reduces the effect of oxidative aging on the bitumen [23]. Practically speaking, this happens when it prevents the increase in the T* upon aging. Therefore, the determination of T* in aged bitumen and its comparison with the value of unaged bitumen can give information about the antioxidant effect of the various types of CS. Table 1 shows also the results of aging processes by reporting the increase in transition temperature (ΔT) after aging, values obtained as the difference between the T* recorded after and before the aging process RTFOT at short (75 min) and long (225 min) times (see third and fourth columns). From these values, it can be seen that the CS content is important in regard to giving such an effect: by increasing the CS content, the ΔT decreases. In addition, at 6 wt%, almost all the samples give a certain antioxidant effect. The most marked effect is exerted by **CS2** and **CS4**.

Also, in this case, the **CS2** polymer proves to be a good additive in the sense that, at a content of 6 wt%

(1) it is able to set the increase in transition temperature at a value of only 4.2 °C after a short period (75 min) of aging;
(2) it sets the increase in transition temperature at a value of 13.2 °C upon a long period (225 min) of aging.

However, the best performance for reducing the long-term aging is given by **CS4,** which is able to set the increase in transition temperature at a value of only 11.4 °C, thus actually reducing the transition temperature increase upon aging of 4.9 °C in comparison to the neat bitumen (NB). The best effect offered by **CS4** could be the consequence of its high molecular weight, which can better reduce the degrees of freedom of the bituminous molecules and consequently their kinetics, with a final slowing down of the overall aging process. It can be therefore argued/hypothesized that the deacetylation grade plays a minor role in this case. This improvement is a positive step for the life cycle of asphalt pavements and can potentially increase the service life of these pavements.

### 2.4. Rigidity at Working Conditions

**Figure 5:**
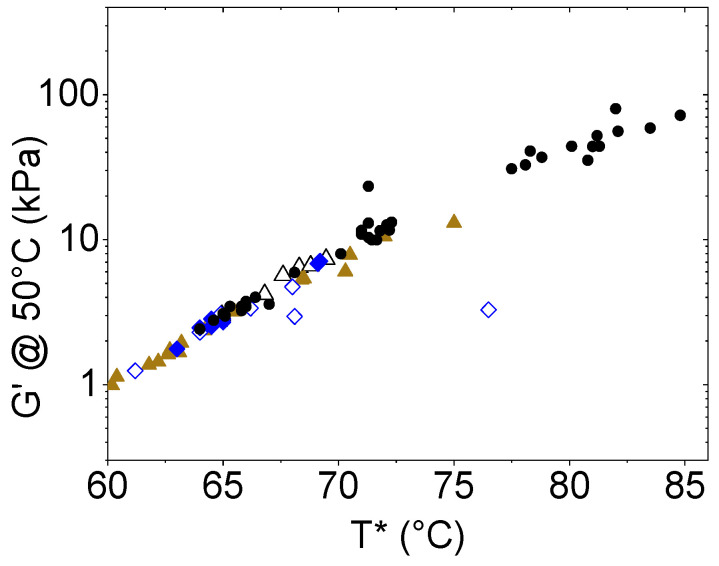
Correlation between G′@50 °C and T* in bitumen reinforced with polysaccharides [25] (brown triangles), with inorganic fine particles [26] (blue diamonds), with char from pyrolysis [27] (empty black triangles) and with cellulose [28] (empty blue diamonds), as compared to the data of this work on CS (black circles).

The above results have been obtained by the analysis of T*. However, it is worth also considering another physical parameter, specifically the G′ value measured at 50 °C (G′@50 °C). This is another important parameter assessing the mechanical property of bituminous materials: it is usually accepted to well represent the rigidity of bitumen at working conditions [24]. We found that G′@50 °C is nicely correlated with T* (see Figure 5). Since they are two independent parameters, their correlation suggests that the conclusions derived by analysis of T* (see Section 3.2 and Section 3.3) can be derived also if G′@ 50 °C is considered, thus reinforcing the hypothesis that CS can be a good modifier and antioxidant agent for bitumen.

Even more, our data are in accordance with literature data from other bituminous samples (see Figure 5, where data from bitumen reinforced with polysaccharides [25], with inorganic fine particles [26], with char from pyrolysis [27] and with cellulose [28] are also reported for comparison both for T* and G′@50 °C).

### 2.5. Adhesion to Stone

**Figure 6:**
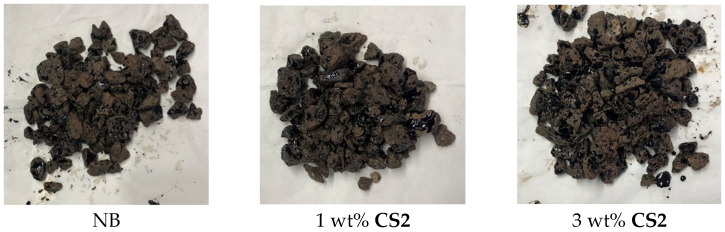
Some representative images of porphyry stones after boiling tests.

To evaluate the feasibility of using these additives, and, in particular, to ascertain that CS does not alter the bitumen adhesion to stones, the adhesion to stone was tested by boiling tests [29,30] using a porphyry as a representative stone [31,32]. The chemical analysis of this type of stone is reported in ref. [33]. Some representative images are reported in Figure 6, where some representative samples are shown. As a result of these tests, no significant changes have been observed. Although the quantitative/precise assessment of loss of chemical adhesion of a bitumen onto a stone would require specific laboratory methodologies, [33] it can, however, be concluded that all additives do not worsen the adhesion property of the binder with this stone; therefore, the adhesive properties of the binder are preserved.

## 3. Materials and Methods

### 3.1. Materials

Four different CS polymers have been used for this work, purchased from two different companies.

**CS1**: from Sigma–Aldrich (St. Louis, MO, USA), low molecular weight (50,000–190,000 kD, with a deacetylation degree ≥75% (free amine groups); **CS2**: from Sigma–Aldrich, molecular weight 310,000–375,000 kD with a deacetylation degree ≥75% (free amine groups); **CS3**: from Acros Organics (Geel, Belgium), molecular weight 100,000–300,000 kD with a deacetylation degree >90% (free amine groups); **CS4**: from Acros Organic, high molecular weight (600,000–800,000 kD) with a deacetylation degree >90% (free amine groups) (Table 2).

The bitumen, sourced from Saudi Arabia, was provided by Lo Prete (Italy). By the standard testing method outlined in ASTM D946, which corresponds to EN 1426, for assessing bitumen and bituminous binders by a needle (loaded with a 100 g weight and inserted into the bitumen for a specified duration at a controlled temperature) penetration [34], it was found that the penetration grade was 50/70. Other characteristics are reported in a previous work [35]. The concentrations (wt%) of the four main bitumen components (saturates, aromatics, resins, asphaltenes), evaluated by the S.A.R.A. method (which essentially exploits the different solubilities of these fractions in opportune solvents [7]), were found to be 3.8, 51.3, 21.5, 23.4, respectively, and are reported in Table 3. Hereafter, the neat, unmodified, bitumen will be labeled as “NT”.

For boiling tests, acidic stone (commercial name: “Porfido del Trentino”) was chosen as testing stone material. It was kindly furnished by the company “Porfido Trentino SRL” (Albiano, Northern Italy). Generally, the chemical composition of this stone is mainly composed of silica, aluminia, alkalis and small percentages of iron, calcium and magnesium, while the mineralogical composition is composed of quartz crystals, sanidine crystals, plagioclase crystals and, to a lesser extent, biotite and pyroxenes.

### 3.2. Bitumen Blend Preparation

CS addition at the desired content (1, 3, 6 wt%) was made to fully flowing hot bitumen (150 ± 10 °C). Mechanical stirring at 500–700 rpm (30 min) was carried out for blend homogenization by an IKA RW20, Königswinter, Germany. These conditions, as for our experience, can ensure the homogeneity of the samples without sample degradation. In any case, this method is a standard operation, coherent with procedures reported by other authors [36]. After mixing, the final bituminous sample was poured into a small sealed can and stored in a dark chamber at 25 °C. Due to attention in making the results comparable, care was taken to always keep the same operation conditions.

### 3.3. Thermogravimetric Analysis

The thermal stabilities of the employed CS polymers were probed by TGA measurements performed on a Perkin–Elmer TGA 8000 Analyzer (PerkinElmer, Inc. 940 Winter Street. Waltham, MA, USA). All thermograms were recorded at a scan rate of 10 °C/min in the temperature range 26–850 °C from ca. 10 mg of each polymer. The thermal stability over time of the employed CS was probed at 150 °C by recording an isotherm analysis at 150 °C for 95 min for all polymers using the same instrument.

### 3.4. Aging Tests

To replicate the aging process of the samples, the Rolling Thin-Film Oven Test (RTFOT) was performed following ASTM D2872-04 [37], which corresponds to UNI EN 12607-1 [38] for evaluating resistance to hardening under heat and air exposure. The test apparatus consists of a double-wall furnace with an internal fan that circulates hot air at a controlled temperature of 163 °C. Inside the oven, eight specially designed glass bottles are positioned horizontally on a rotating carousel. During the test, a thin bitumen layer of approximately 1.25 mm is exposed to a stream of hot air for either 75 or 225 min. The extended duration of 225 min was selected to simulate prolonged aging for comparison with the standard 75-min period. Each modified bitumen sample, along with a reference binder without additives, was divided into two portions, with only one undergoing artificial aging. Table 1 presents the data comparing the properties of the samples before and after the aging process.

### 3.5. Rheological Tests

The rheological analysis involved determining the complex shear modulus [39], expressed as G* = G′ + i G″, as a function of temperature using dynamic shear rheometer (DSR) testing. The measurements were conducted with a dynamic stress-controlled rheometer (SR5000, Rheometric Scientific, Piscataway, NJ, USA) featuring a parallel plate configuration (2 mm gap, 25 mm diameter) at a fixed frequency of 1 Hz. These testing conditions aligned with previous reported research studies [40]. Temperature regulation was maintained by a Peltier element with an accuracy of ±0.1 °C, and the heating rate was set to 1 °C/min. To ensure linear viscoelastic behavior, preliminary stress sweep tests were performed and small-amplitude oscillatory shear conditions were maintained throughout the measurements. 

## 4. Conclusions

In conclusion, the study advocates for the addition of chitosan (CS) into bitumen as a viable method to enhance the performance and sustainability of road construction materials. In particular, the following clues can be reported:

- Best results in terms of rheological modifier were obtained for CS with a molecular weight of 310,000–375,000 kD and with a deacetylation degree ≥75% (free amine groups);
- A good antioxidant effect was shown by CS with high molecular weight (600,000–800,000 kD) and with a deacetylation degree >90% (free amine groups);
- No detrimental effect on the adhesion efficiency with the stones was observed, suggesting the possible use of CS as an additive;
- The findings suggest that CS can play a critical role in developing more durable, environmentally friendly, and cost-effective bitumen formulations, since CS is readily available from the waste shells of crustaceans obtained from seafood processing industries, making it a potentially sustainable and environmentally friendly material. The utilization of waste materials for CS extraction may present economic benefits by valorizing underutilized resources and reducing waste disposal costs; ultimately, it can represent a sustainable approach with significant potential to contribute to the circular economy.

This paper calls for further research to optimize the use of CS in bitumen and to fully understand its long-term effects on road performance. It must be noted that the evaluation of the performance of this additive should evolve towards verifying its effect on asphalt mixtures (stones plus bitumen). Ongoing activities are dedicated to assessing the feasibility of recycling CS from food industry waste as a cost-effective and sustainable additive for innovative road pavement design. Notably, a recent study demonstrated the advantages of chitosan-modified road bitumen after de-icing salt treatment in terms of improved resistance to erosion [41]. Combined with our findings, these studies provide strong evidence that chitosan (CS) can serve as a sustainable approach to road construction. 

## Figures and Tables

**Table 1:** Transition temperature (T*) of the bituminous samples. The highest effects are highlighted by bold characters.

Sample	T* (°C)	ΔT*_1_ = T*_75min_ − T*_unaged_	ΔT*_2_ = T_225min_ − T_unaged_
Neat Bitumen (NT)	64.6	5.5	16.4
1wt% **CS1**	65.1	6.55	15.0
3 wt% **CS1**	66.4	4.9	11.7
6 wt% **CS1**	64.0	7.45	14.8
1 wt% **CS2**	65.1	7.0	18.4
3 wt% **CS2**	65.0	6.0	17.0
6 wt% **CS2**	68.1	4.2	**13.2**
1 wt% **CS3**	65.3	6.8	19.5
3 wt% **CS3**	66.0	5.8	14.8
6 wt%**CS3**	67.0	4.3	14.2
1 wt% **CS4**	65.8	6.4	16.3
3 wt% **CS4**	65.8	5.5	12.5
6 wt% **CS4**	66.0	5.0	**11.5**

**Table 2:** Molecular weight and deacetylation degree for the four CS polymers used.

Modifier	Molecular Weight (kD)	Deacetylation Degree
**CS1**	50,000–190,000	≥75%
**CS2**	310,000–375,000	≥75%
**CS3**	100,000–300,000	>90%
**CS4**	600,000–800,000	>90%

**Table 3:** Essential properties of the bitumen used.

Property	Value
origin	Saudi Arabia
penetration grade	50/70
saturates content (wt%)	3.8
aromatics content (wt%)	51.3
resins content (wt%)	21.5
asphaltenes content (wt%)	23.4

## Data Availability

Data are contained within the article.
